# Epidemiology and seroepidemiology of human enterovirus 71 among Thai populations

**DOI:** 10.1186/1423-0127-21-16

**Published:** 2014-02-18

**Authors:** Piyada Linsuwanon, Jiratchaya Puenpa, Sheng-Wen Huang, Ya-Fang Wang, John Mauleekoonphairoj, Jen-Ren Wang, Yong Poovorawan

**Affiliations:** 1 Center of Excellence in Clinical Virology, Department of Pediatric, Faculty of Medicine, Chulalongkorn University, Bangkok 10330, Thailand; 2 Department of Medical Laboratory Science and Biotechnology; Center of Infectious Disease and Signaling Research, National Cheng Kung University; National Institute of Infectious Diseases and Vaccinology, National Health Research Institutes, Tainan 701, Taiwan

**Keywords:** Hand foot and mouth disease, Enterovirus epidemiology, Seroprevalence, Human enterovirus 71

## Abstract

**Background:**

Human enterovirus 71 (EV71) is an important pathogen caused large outbreaks in Asian-Pacific region with severe neurological complications and may lead to death in young children. Understanding of the etiological spectrum and epidemic changes of enterovirus and population’s immunity against EV71 are crucial for the implementation of future therapeutic and prophylactic intervention.

**Results:**

A total of 1,182 patients who presented with the symptoms of hand foot and mouth disease (67.3%) or herpangina (HA) (16.7%) and admitted to the hospitals during 2008-2013 were tested for enterovirus using pan-enterovirus PCR targeting 5′-untranslated region and specific PCR for viral capsid protein 1 gene. Overall, 59.7% were pan-enterovirus positive comprising 9.1% EV71 and 31.2% coxsackievirus species A (CV-A) including 70.5% CV-A6, 27.6% CV-A16, 1.1% CV-A10, and 0.8% CV-A5. HFMD and HA occurred endemically during 2008-2011. The number of cases increased dramatically in June 2012 with the percentage of the recently emerged CV-A6 significantly rose to 28.4%. Co-circulation between different EV71 genotypes was observed during the outbreak. Total of 161 sera obtained from healthy individuals were tested for neutralizing antibodies (NAb) against EV71 subgenotype B5 (EV71-B5) using microneutralization assay. The seropositive rate of EV71-B5 was 65.8%. The age-adjusted seroprevalence for individuals was found to be lowest in children aged >6 months to 2 years (42.5%). The seropositive rate remained relatively low in preschool children aged > 2 years to 6 years (48.3%) and thereafter increased sharply to more than 80% in individuals aged > 6 years.

**Conclusions:**

This study describes longitudinal data reflecting changing patterns of enterovirus prevalence over 6 years and demonstrates high seroprevalences of EV71-B5 NAb among Thai individuals. The rate of EV71 seropositive increased with age but without gender-specific significant difference. We identified that relative lower EV71 seropositive rate in early 2012 may demonstrate widely presented of EV71-B5 in the population before account for a large outbreak scale epidemic occurred in 2012 with due to a relatively high susceptibility of the younger population.

## Background

Human enterovirus 71 (EV71) is a group of genetically diverse viruses belonging to the genus *Enterovirus* species A, family *Picornaviridae*. EV71 was first isolated from an infant with encephalitis in California in 1969, but retrospective studies in the Netherlands using the clinical specimens collected in 1963 [1] combined with evolutionary analysis suggest that EV71 could have emerged as early as 1941 [2]. Since then, the virus has spread worldwide and has been implicated as the predominant causative pathogen in the outbreaks of hand, foot, and mouth disease (HFMD) and herpangina (HA). Infection by EV71 is often asymptomatic, but may manifest as self-limiting disease with mild flu-like symptoms. However, in some cases, EV71 infection may cause polio-like myelitis, brainstem encephalitis, aseptic meningitis, and even death resulting from complications [3-9]. Mortality rates due to EV71 infection in the Asian-Pacific countries ranged from < 0.5% and up to 19% [10-14]. Although infection by other enteroviruses can also contribute to HFMD and HA with similar clinical manifestation as EV71, the symptoms are milder with a much lower incidence of severe complications [15-17]. Therefore, early and accurate detection of EV71 in clinical settings is considered a medical necessity.

EV71 is an icosahedral encapsulated particle, which comprises a positive-sense single-stranded RNA genome of approximately 7.4 kb in length. The linear genome is organized in a single long open reading frame (ORF) flanked by the 5′ and short 3′-untranslated regions (UTR) with a polyadenylated tail. The ORF encodes a polyprotein which is processed to form the four mature structural capsid proteins (VP1-VP4) and seven non-structural proteins (2A-2C and 3A-3D) responsible for viral replication and protein processing. VP1 is the immune-dominant capsid protein and contains the most important neutralization epitopes. It shows a sequence divergence and has been used for molecular type assignment and evolutionary study. On the basis of the molecular characterization of VP1, EV71 can be phylogenetically classified into three genotypes (A, B, and C). Genotypes B and C can be further divided into subgenotypes B1-B5 and C1-C4, respectively. Genotypes B and C are distributed worldwide and exhibit distinct evolutionary characteristics, while genotype A is much less common [18,19]. EV71 is likely transmitted via oral-fecal route and is possibly shed from the respiratory tract via aerosolized droplets. EV71 is highly contagious and easily infects persons in close contact with other infected individuals. The incubation period for EV71 is assumed to be approximately 5 days (d) with the median duration of virus shedding period estimated to be 2 d in close contact group, 18 d in mild HFMD and 25 d for severe HFMD [20]. The median basic reproductive number for EV71 is 5.48, which is approximately two-fold higher than that of coxsackievirus (CV) type A16 infection (2.50 d) [21].

Since the late 1990s, EV71 infection has been documented in many Asian countries [11,22-28]. Therefore, HFMD has been included in the communicable disease reporting system of the Thai Ministry of Public Health since 2001. Reports from the ministry revealed that the number of sporadic HFMD cases in Thailand during 2007 to 2011 was approximately 12,000 to 18,000 cases annually. However, a large-scale outbreak of HFMD was first reported on July 19, 2012. This outbreak affected more than 39,000 patients with an estimated incidence of 62.7/100,000 person-years during the outbreak period [29] and was the highest in a decade. By the end of July, three subsequent deaths were reported. Of those, one case was a girl aged > 2 years 8 months from Bangkok who had a history of frequent asthma exacerbation. Another case involved a 2 years old boy from a Cambodian migrant family who had been living in Rayong province. Both had been diagnosed with HFMD with respiratory failure and died soon after hospitalization, but without neurological complications. In addition, a 16 years old boy in Sakaeo province had been diagnosed with HFMD with brain encephalitis and succumbed to heart and respiratory system failure. Laboratory tests on throat swab specimens implicated EV71 subgenotype B5 (EV71-B5) in all these fatal cases. Although the epidemiology and seroepidemiology of EV71 and other enteroviruses have been well-studied in many countries, limited surveillance data are available in Thailand. In this study, we analyzed the enteroviral etiology associated with HFMD and HA in Thailand from 2008 to 2013 and aimed to identify evidence that could explain the sudden outbreak of HFMD and HA in 2012. We also assessed the Thai population’s immunity against EV71-B5 using archived sera collected before the large outbreak in 2012. The knowledge gained will be useful in understanding the viral genetic diversity and selection of effective vaccine strains for prevention.

## Methods

### Ethical consideration

The study protocols were approved by the institutional review board of the Faculty of Medicine, Chulalongkorn University, Thailand. The epidemiological study was conducted on clinical specimens collected upon conclusion of routine examinations and stored as anonymous. Serum samples were obtained from healthy individuals from two previous studies (influenza and biliary atresia control group) [30,31]. Patient identifiers including personal information (name, address) and hospitalization number were removed from these samples to protect patient confidentiality. Since the data obtained for this study were de-identified, written consents were waived. Permission for specimen utilization had been granted by the Director of King Chulalongkorn Memorial Hospital. The research protocols for prospective molecular epidemiological and seroepidemiological studies were approved under protocol numbers IRB390/55 and IRB068/56, respectively, and conducted in compliance with the principles of the Declaration of Helsinki.

### Case definition and data collection

Case definitions for the diseases are as followed: patient with HA was defined as having well-characterized vesicular enanthem, oral ulcers on the anterior tonsil pillars, soft palate, buccal mucosa, or uvula. Patients were diagnosed with HFMD if they had oral ulcers mainly on the buccal mucosa and tongue, accompanied by typical vesicular rashes most commonly on the extensor surfaces of the hands, feet, knees, and/or buttock. A severe case was defined as having HFMD or HA and accompanied by the occurrence of at least one of the following complications: aseptic meningitis, myocarditis, encephalitis, pulmonary oedema, haemorrhage, acute flaccid paralysis, and cardiopulmonary collapse [11,32]. Additionally, patients could be classified as having severe HFMD or HA whether or not they experienced nervous system complication.

### Clinical specimen and data collection

**Figure 1:**
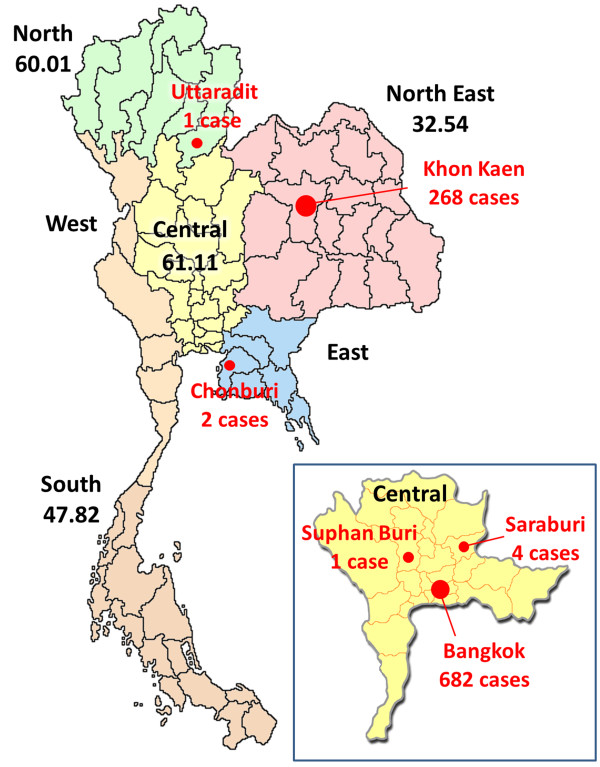
**Geographic distribution of the study population during the HFMD outbreak in 2012 in Thailand.** Closed circles on the map indicate the locations and numbers of patients collected during the outbreak period (red). Data of the estimated numbers of cases per 100,000 person-year in different regions (if available) were obtained from the Thai Ministry of Public Health of Thailand and are indicated under the geographic region (black).

Clinical specimens were collected from patients who met the inclusion criteria and information on patient demographics (age, sex and hospital location), clinical symptoms, discharge diagnosis and the date of sample collection were recorded using a standardized form. All specimens were then transported within 48 hours to the Center of Excellence in Clinical Virology, Chulalongkorn Hospital, for causative pathogen identification. During the period from 2 June 2008 through 3 October 2013, we received 1,239 clinical specimens obtained from 1,182 in- and outpatients who presented signs and symptoms of HFMD or HA and were admitted to hospitals and medical centers in several parts of Thailand. Among these, 224 cases were collected from the Pediatrics Department of Chulalongkorn Hospital in Bangkok between 2008 and 2013. Additional 682 cases were from 17 hospitals and medical centers located in Bangkok. Another 276 cases were from collaborative provincial tertiary hospitals in 5 different provinces including 268 cases from KhonKaen (northeastern Thailand), 4 cases from Saraburi (central Thailand), 2 cases from Chonburi (eastern Thailand), and 1 case each from SuphanBuri (central Thailand) and Uttaradit (northern Thailand) (Figure 1). All of the cases outside of Chulalongkorn Hospital were received during the outbreak in 2012 and 2013. Types of clinical specimens are as followed: of all 1,239 samples, 822 were rectal swabs, 196 were stool samples, 123 were throat swabs, 35 were blood components (serum, EDTA and clotted blood), 18 were cerebrospinal fluids, 17 were vesicle fluids and swabs, 14 were nasal swabs, 5 were nasopharyngeal suctions, 3 were sputum samples, 2 were nasal washes, 3 were mouth ulcer tissues, and 1 was a saliva sample.

### Enterovirus screening and molecular typing by phylogenetic analysis

Total virus nucleic acid and molecular typing of enterovirus from samples were previously described [33,34]. Briefly, pan-enterovirus (pan-EV) primers were employed for enterovirus detection by amplifying a target site within the highly conserved 5′-UTR using semi-nested polymerase chain reaction (PCR). To further confirm the enterovirus type, CODEHOP degenerate primers targeting the VP1 gene of all enterovirus types were used [35]. Different sets of specific primers targeting the VP1 gene for all genotypes and/or types of EV71, CV-A16, and the current disseminating CV-A6 viruses were employed [33]. The amplified products were purified using the HiYield Gel DNA Fragment Extraction kit (RBC Bioscience, Taiwan). All sequencing was performed bi-directionally using the primers of the 2^nd^ round PCR by an automated cycle DNA sequencer (First BASE Laboratories, Malaysia).

The nucleotide sequences were edited using the Seqman program of DNASTAR Software (v5.0). Sequences were subjected to BLAST analysis and aligned using the multiple alignment program MAFFT (v7.0) (http://mafft.cbrc.jp/alignment/server/index.html) followed by manual editing with BioEdit program (v7.1.9) (http://www.mbio.ncsu.edu/bioedit/bioedit.html). In order to classify enterovirus types, which were directly identified from the clinical specimens, neighbor joining phylogenetic analysis was performed. The genetic distances were calculated according to the maximum composite likelihood model with 1,000 bootstrap pseudo-replicates and pairwise deletion for missing data implemented in MEGA software (v5.0) (http://www.megasoftware.net/). The nucleotide sequences obtained from this study have been deposited in the GenBank database under accession numbers GQ449258-GQ449279, JX556607-JX556655, and KF661098- KF661107.

### Experimental serum samples

The samples were cross-sectionally stored sera collected between 2009 and July 2012 (prior to the outbreak) from healthy individuals of different age groups who had participated in previous serosurveillance studies, including volunteers and medical personnel in Bangkok and KhonKaen [30,31]. None of the participants presented any symptoms of HFMD or HA at the time of serum collection. The stored data included age band, gender, locality or medical centers, any recorded symptoms or clinical information, referral source, the month of sample collection, and the results of other virology tests for each sample (when available). In order to determine the age-related seroprevalence of EV71 neutralizing antibody (NAb) against EV71-B5, 161 subjects were categorized into six age groups (infants aged ≤ 6 months, children aged > 6 months to 2 years, pre-elementary school children aged > 2 years to 6 years, elementary school children aged > 6 years to 12 years, adolescents aged > 12 years to 18 years, and adults aged > 18 years).

### NAb detection by epitope-blocking ELISA assay

The NAb titer was measured by using rhabdomyosarcoma (RD) cell based microneutralization-ELISA assay. The virus strain used in this study was EV71-B5 designated EV71/Taiwan/1745/08 (FJ357352), which shared > 99% amino acid sequence to the previously defined Thai strains based on both whole genome and VP1 sequence analysis [36]. The diluted serum samples were mixed with an equal volume of 200 tissue culture half-infective dose of the virus, and then incubated at 37°C for 2 hours. After the incubation period, the antibody-virus mixtures were added to the microtiter plates containing 1.5 × 10^5^ cells/ml RD cells and subsequently incubated in a 5% CO_2_ incubator at 37°C for 24 hours. The EV71 virus antigen was detected by ELISA and the optical density was read at a primary wavelength of 450 nm using a microplate reader. The standard NAb level, which reflects defensive capacity against EV71, has not been assigned. We therefore defined a cutoff value of 1:8 to be seropositive, similar to the previous serosurveillance studies and preclinical evaluation of the candidate vaccine [37]. The highest dilution of serum samples that inhibited virus growth and prevented the occurrence of cytopathic effect was considered the neutralization antibody titer. The cutoff values for data interpretation were 1:8-1:64, 1:128-1:256, and ≥ 1:512 (low-, medium-, and high-NAb titers, respectively). Each serum specimen was tested in duplicate to reduce assay variation.

### Statistical analysis

Statistical data comparisons between various factors were analyzed by means of Pearson χ^2^, unpaired T-test, or Fisher’s exact test as appropriate using SPSS software (v17.0) (Chicago, USA). All data were considered statistically significant at a *p*-value below 0.05. The geometric mean titer (GMT) of positive sera and the corresponding 95% confidence intervals (CI) were computed by taking the log-transformation of the titer values, followed by antilog-transformation.

## Results

### Epidemiological data of study population

**Figure 2:**
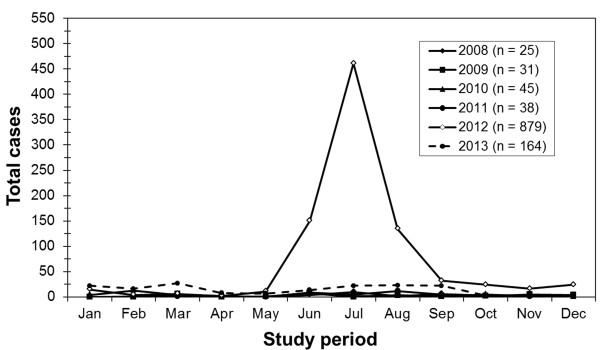
**The distribution of HFMD and HA cases for each month between the years 2008 and 2013.** An outbreak occurred between June and August in 2012.

During the study period, a total of 1,239 clinical specimens from 1,182 patients with confirmed or suspected diagnosis of HFMD or HA were included. The number of monthly cases for each year between 2008 and 2013 are shown in Figure 2. While fewer than 40 cases were observed between 2008 and 2011, an outbreak occurred in 2012, which peaked in July of that year. Since the specimens were sent to our Center as anonymous samples, redundancy due to multiple samplings or follow-up visits by the same individuals could not be investigated. The majority of the cases presented mild forms of HFMD or HA. A total of 67.3% (795/1182) and 16.7% (197/1182) of all cases presented with HFMD and HA, respectively, whereas 16.1% (190/1182) had incomplete data on the clinical diagnosis (Table 1). The remaining unspecified cases were categorized as the HFMD/HA group. The average age of patients in conjunction with the respective disease are also shown in Table 1. Among the study population, 123 cases (10.4%) are of unknown age (37 cases of HFMD, 5 cases of HA, and 81 cases of HFMD/HA). The overall age distribution of enrolled patients was between 1 d and 54 years old (y). The mean age was 3.6 years ± 5.0 years and median age was 2.5 years. There were no statistical differences with regards to male-to-female ratio among patients in the different disease groups.

**Table 1:** Demographic and clinical data associated with different enterovirus genotypes

	**HFMD**	**HA**	**HFMD/HA**	**All**
No of case	795 (67.3%)	197 (16.7%)	190 (16.1%)	1182
No of sample	833 (67.3%)	202 (16.4%)	202 (16.4%)	1237
Age range (median)	4d to 54y (2.4y)	3 m to 16y (2.6y)	1 m to 50y (3y)	4d to 54y (2.5y)
Mean age ± SD	3.4y ± 4.8y	3.4y ± 3.0y	5.1y ± 8.3y	3.6y ± 5.0y
Sex ratio (F:M)	1:1.4	1:1.1	1:1.2	1:1.3
Virology: EV71	66 (8.3%)	2 (1%)	40 (21.1%)	108 (9.1%)
CV-A16	65 (8.2%)	2 (1%)	35 (18.4%)	102 (8.6%)
Other CV-A	217 (27.3%)	22 (11.2%)	28 (14.7%)	267 (22.6%)
Pan-EV	131 (16.5%)	88 (44.7%)	10 (5.3%)	229 (19.4%)
Negative	316 (39.7%)	83 (42.1%)	77 (40.5%)	476 (40.3%)

HFMD, Hand, foot, and mouth disease; HA, Herpangina; EV71, Human enterovirus 71; CV-A, Coxsackievirus species A; Pan-EV, Pan-enterovirus; y, year old; m, month old.

### Genotype change of enteroviruses in Thailand during 2008 to 2013

**Figure 3:**
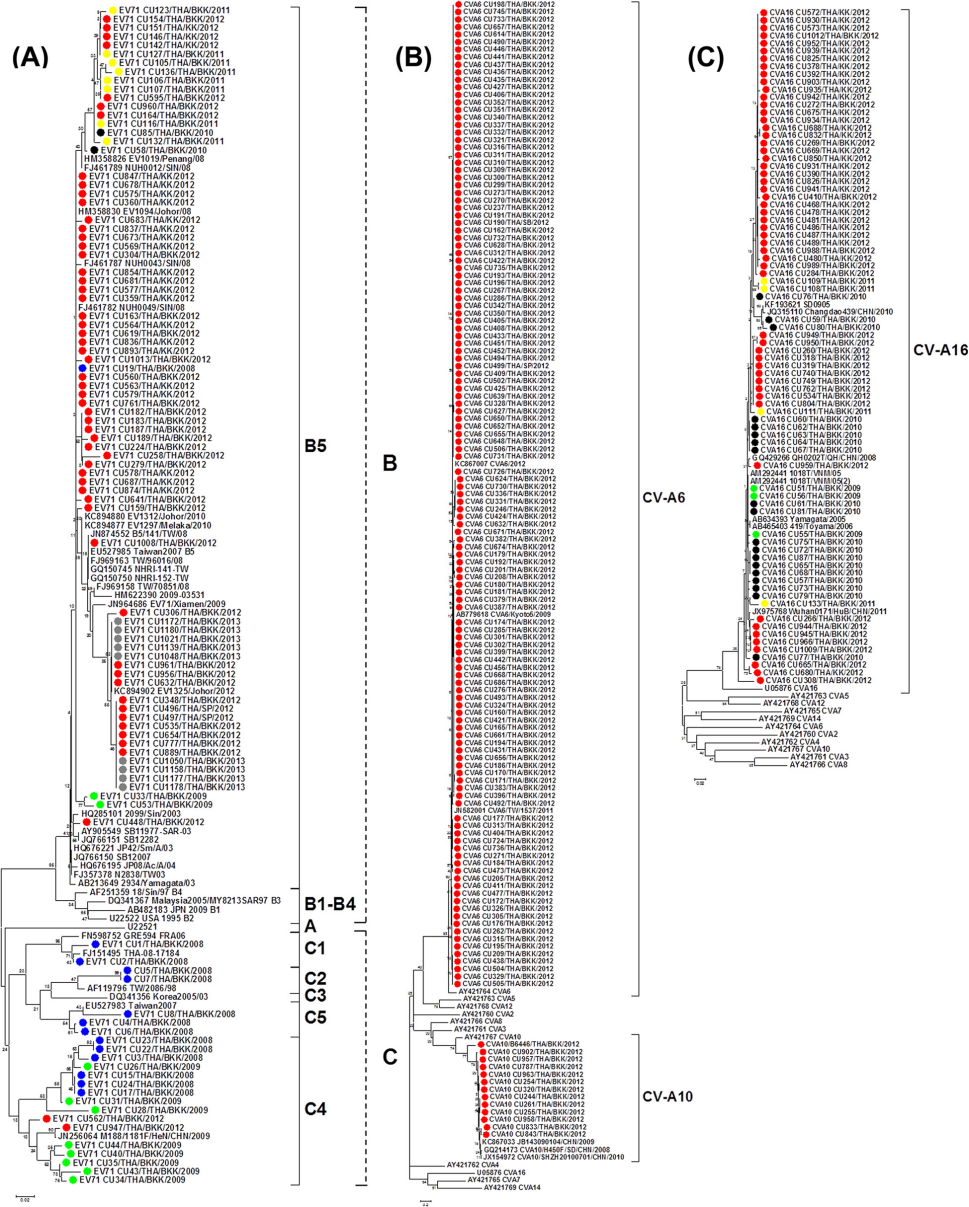
**Neighbor-joining estimates of the phylogenetic relationship for enteroviruses detected among Thai pediatric children during 2008-2013.** The trees were generated on the basis of partial VP1 nucleotide sequence data. Virus strains characterized in this study are indicated with colored dot according to year of sample collection and with species as the followings: **(A)** EV71, **(B)** CV-A6 and CV-A10 and **(C)** CV-A16. Dash lines indicate subgenotype of EV71 and vertical lines indicate subgenotypes of EV71 and types of CV-A. Scale bar indicates the number of nucleotide substitutions per site.

Using the standard semi-nested pan-EV RT-PCR assay, we identified enteroviruses in 59.7% (706/1,182) of all the cases (Table 1). Phylogenetic analysis of VP1 sequences obtained from this study along with others from the GenBank database a total of 477 sequences examined showed that 9.1% were positive for EV71 and 31.3% were various CV types (Figure 3A). The latter was further categorized as EV-A species, including 70.5% CV-A6 (260/369), 27.6% CV-A16 (102/369), 1.1% CV-A10 (4/369), and 0.8% CV-A5 (3/369) with bootstrap values higher than 95% (Figures 3B-C).

**Figure 4:**
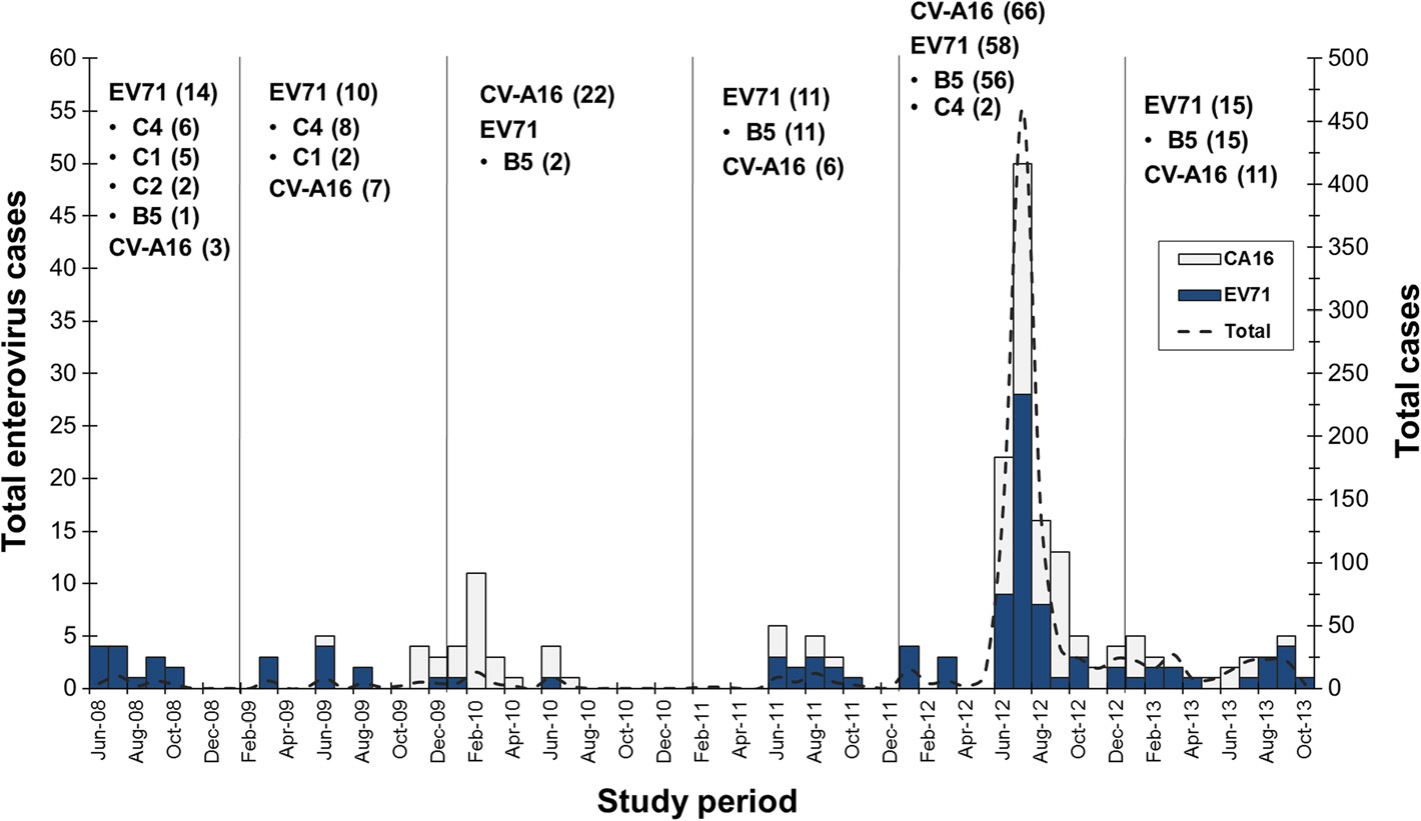
**Successive appearance and distribution of predominant types and genotypes of enterovirus between 2008 and 2013.** Numbers of cases are indicated after types and genotypes of viruses.

**Figure 5:**
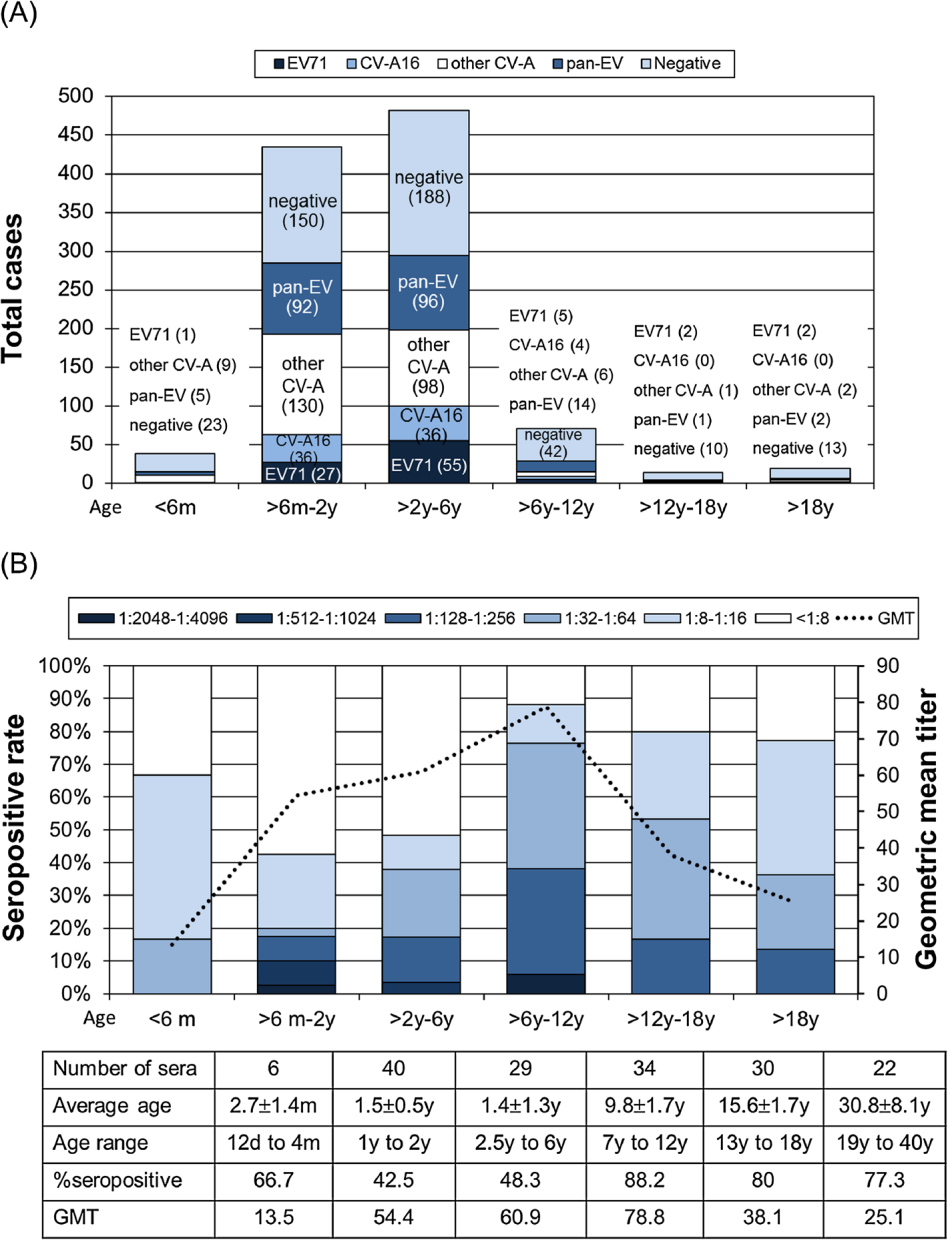
**Distribution of types of enterovirus and serum NAb against EV71 subgenotype B5 in the study population. (A)** Age distribution of enterovirus infected patients with HFMD and HA during the study period and. Bar colors correspond to the numbers of patients with different types of enterovirus identified during the study period are indicated in the graph. **(B)** Serum titer of NAb against EV71 subgenotype B5 relative to age. The seropositive rates of individuals with NAb titers of 2-fold dilution are plotted on the y-axis. The dash line denotes the cumulative geometric mean titer according to donors’ age. The number of sera (161 total) from each of the six age groups are indicated in the table below the graph.

Figure 4 shows the distribution of the predominant genotypes detected throughout the study period. Both EV71 and CV-A16 were predominant in HFMD cases compared to HA cases (*p* < 0.05). Age distribution of the enterovirus infected patients is shown in Figure 5A. HFMD patients infected by CV-A16 (2.7 years ± 1.5 years with a median age of 2.5 years) were generally younger than those infected by EV71 (3.4 years ± 3.4 years with a median age of 2.6 years) and the non-EV infected group (4.4 years ± 3 years with a median age of 6.3 years). However, these differences were not statistically significant. When we compared the correlation between the clinical diagnosis and the different viral infection, the result showed that patients with HFMD diagnosis had a higher rate of EV71 infection (61.1% (66/108)) compared to those with HA (1.9% (2/108)) and HFMD/HA (37% (40/108)).

Between 2008 and 2011, a total of 139 sporadic and endemic cases of HFMD and HA in Bangkok were examined. Most enteroviruses detected belonged to genotypes within the enterovirus species A (60.4% (84/139)). EV71 was the most prevalent among the endemic cases, while CV-A16 was not found. In 2008, EV71 and CV-A6 were detected in 56% (14/25) and 12% (3/25), respectively. Molecular typing and phylogenetic analysis based on VP1 sequences of EV71 revealed the circulation of various genotypes of EV71 including 6 strains of EV71-C4, 5 strains of EV71-C1, 2 strains of EV71-C2, and 1 strain of EV71-B5 (Figure 4). In 2009, 32% (10/31) and 23% (7/31) of cases showed EV71 and CV-A16 infection, respectively. The EV71 genotype C4 was the most prevalent (8/10), followed by genotype B5 (2/10). Other enterovirus types were also detected including one each of CV-A10 and CV-A6. In 2010, CV-A16 became the predominant virus, constituting 48.9% (22/45) of all cases. EV71 was detected only in 2 cases and both were B5 subgenotype. CV-A6 was sporadic with a total number of 4 cases (8.9%). In 2011, EV71 (29% (11/38)) predominantly replaced CV-A16 (16% (6/38)), while EV71-B5 was the only subgenotype identified suggesting a continuous circulation of EV71-B5 in Thailand. Of these, 2 and 1 cases were diagnosed positive for CV-A10 and CV-A6, respectively.

During January to February 2012, only EV71 was identified among the study population (36.8% (7/19)). The following month, this EV71 dissemination was replaced by the infrequent CV-A6, which then circulated until the end of the year. The number of cases sent to our Center dramatically increased beginning in June 2012. Of the 879 clinical specimens, CV-A6 was the predominant virus and accounted for 28.4% of the cases (250), followed by 6.9% CV-A16 (61), 0.5% for other minor enterovirus species A (4) and 22.4% for untypable pan-EV (197). It is noteworthy that EV71 accounted for only 6.6% (58/879) even though it was predominant in the previous years. Co-circulation between different EV71 genotypes was also observed. Among those, 96.5% of EV71 belonged to subgenotype B5 (56/58), while only 3.5% (2/58) were subgenotype C4. Among confirmed patients, the average age during the large outbreak was 3.1 years ± 4.3 years with a median age of 2.3 years. These data therefore suggest that HFMD and HA frequently occurred in young children. Most of the cases presented with mild HFMD symptoms (77.1% (678/879)), and mild HA cases accounted for 18.8% (165/879). During the outbreak, there were 6 mild HFMD patients who had presented with distinct diseases including 3 cases with acute febrile illness diagnosis and 1 case each with abdominal pain, exudative tonsillitis, and upper respiratory tract illness. Two severe HFMD with myocarditis and complex febrile convulsion were observed. The median age of all of these patients was 3.2 years. However, all cases tested negative by both pan-EV and specific PCR screening. Analysis of the clinical manifestations showed that patients infected with CV-A6 had a higher rate of skin vesicles and ulcers particularly in the perianal area, knees and elbows than patients infected with EV71 and CV-A16. However, no significant difference was found.

In 2013, EV71 and CV-A16 were detected in 9% (15/164) and 7% (11/164), respectively. Although no large scale outbreak occurred in 2013, these rates are similar to those reported in the previous years. Among the EV71 infection cases, B5 was the only subgenotype identified. As for the seasonal distribution of HFMD and HA cases attributable to enteroviruses, it mainly occurred in two annual periods (between June and October in 2008, 2011 and 2012 and between January and July in 2010). Furthermore, HFMD and HA were found throughout the year in 2013. The two annual epidemic peaks correspond to the overall enterovirus circulation consistent with previous years.

### Seroprevalence against EV71-B5

The analysis of the NAb titers against EV71-B5 in six age groups is shown in Figure 5B. The overall male-to-female ratio was 1:1.27. The measured NAb levels were as follows: 67.9% (72/106), 26.4% (28/106), and 5.7% (6/106) corresponding to low, medium and high NAb titer, respectively. Serology showed that of the six infants aged ≤ 6 months, four had a high seropositive rate (66.7%). The age-specific seroprevalence was 42.5% (17/40) in children aged > 6 months to 2 years and remained relatively low in preschool children aged > 2 years to 6 years at 48.3% (14/29). There was no statistically significant difference in seropositive rates either in children aged 6 months to 2 years or in children aged 2 years to 6 years (*p* = 0.008). However, there was a significant increase in the mean seropositive rate (88.2%) in elementary school children aged > 6 years to 12 years compared to preschool children (*p* = 0.001). Seropositive rate for EV71 remained steady at approximately 78.8% (41/52) in children > 12 years of age, indicating an increase among older children and adults compared to younger children. To analyze the immunity level, GMT for EV71 NAb was tested. The overall GMT for EV71-B5 was 46.2 with 65.6 (95% CI). Although the sample number is low, our result suggests that the NAb titer distribution pattern was significantly lower in infants aged ≤ 6 months (GMT of 13.5). GMT appeared to be highest at 78.8 among children aged 6 years to 12 years. The GMT distribution was lower in children 12 years to 18 years (GMT of 38.1) similar to that observed in adults > 18 years (GMT of 25.1). Although the study population is relatively small and should be regarded as preliminary, this is the first seroepidemiological characterization of EV71 in Thailand.

## Discussion

HFMD and HA outbreaks associated with EV71 infection continue to pose an enormous public health problem with increasing morbidity and mortality in the Asian-Pacific region over the past decades. The prognostic factor for pandemic spread is currently unknown. Some studies have shown that EV71 dissemination correlates with climate temperature and relative humidity [38,39]. Currently, neither antiviral drugs nor approved prophylactic vaccines against the predominant EV71 are available. Candidate EV71 vaccines in Taiwan and China are now in clinical trials [40-42]. Our study demonstrated continuous inter- and intratypic shifts among the EV71 outbreaks. Not only does the highly contagious EV71 displays a rapid evolutionary change, but multiple types of enterovirus such as CV-A16, CV-A6, CV-A10, CV-B2, and echovirus 30 also have the potential to cause epidemics [43-45]. This heterogeneity has hampered the disease control and could prolong the persistence of diseases in countries where multiple enterovirus strains co-exist. It also highlights the necessity to develop an effective vaccine that elicits strong cross-neutralizing antibody response against the different genotypes of enterovirus and to establish an effective immunization protocol. Furthermore, since Asian countries do not have a coordinated enterovirus surveillance program, collaborative international networks and proactive public health policies are urgently needed. Targeted vaccination of the population at risk especially based on the age-group and incidence rate of EV71 infection described in this study will be useful in the consideration for future immunization strategy.

Epidemiological surveillances showed that outbreaks of EV71 occurred cyclically every 2-3 years and virus infections predominantly affect young children, particularly those younger than 3-5 years of age [46,47]. Previous serosurveillance studies have suggested that the EV71 cyclical pattern is due to the proportion of herd immunity among the susceptible children after a major outbreak [48,49]. The first international report on EV71-associated with HFMD in Thailand was documented in 2011 using stored clinical samples collected since 2008 [34]. Another report utilized cell culture-based method to identify the causative agent associated with an HFMD outbreak in a hospital nursery unit [50]. Although this method is generally reliable, it is labor-intensive, time-consuming, and may fail to identify some types of virus. Not surprisingly, only a small number of cases tested positive. For these reasons, we used a highly sensitive specific PCR technique for virus gene amplification in order to genetically characterize the causative pathogens and to estimate the virus activities associated with HFMD and HA in Thailand from 2008 to 2013.

Considerable insights were gained into the evolution and molecular epidemiology of circulating strains of enterovirus on the basis of the VP1 gene sequence analysis. Published data on the enterovirus epidemiology revealed that the shifts between EV71 genotypes B and C or between EV71 and other types of CV-A occurred simultaneously in Asian-Pacific countries [11,22-27]. In agreement with previous findings, we demonstrated that a change in the prevailing subgenotypes and types occurred over time including the shift of EV71 to CV-A, which occurred in 2010 (EV71 subgenotype C4 to CV-A16) and 2012 (EV71-B5 to CV-A6). Strains of EV71-B5 have caused large outbreaks in Asian countries since 1997 including epidemics in Malaysia, Brunei, Singapore, and Taiwan. In addition, enteroviral infection appears to be increasing in recent years, with the continual emergence of the subgenotype C4 in China since 1998 [23,51-55]. In contrast, epidemiological studies in Europe, the United States, and Australia have identified the circulation of different subgenotypes including B1, B2, C1 and C2 [56-61]. Our study also revealed that the reemergence of EV71 subgenotype B5 occurred in Thailand since 2010 and the virus strains identified during this period were phylogenetically closer to viruses circulating in Malaysia, Singapore and Taiwan between 2008 and 2012. Therefore, international spread of emerging genetic variants into new geographical areas seems to be common and may contribute to large scale epidemics. Studies have shown that EV71-B5 strains isolated from the large outbreak in Taiwan in 2008 are related to those circulating in neighboring countries [23,62,63]. Further correlation between the reemergence of EV71 and transmission from neighboring countries will require additional evolutionary and phylogeographic studies.

We demonstrated that EV71 and CV-A16 are endemic in Thailand since 2008. In mid-2012, Thailand experienced the first large scale outbreak of HFMD and HA in several parts of the country particularly in the central (61.11 cases in 100,000) and the northern region (60.01 cases in 100,000). The initial outbreak began when the number of suspected HFMD and HA cases sent to our Center dramatically increased in June 2012. As a result, we received about 25 times more samples than the previous year (879 cases in 2012 compared to 25-46 samples during 2008-2011). While samples positive for EV71 and CV-A16 were relatively low in most years, we demonstrated that approximately 43% of enterovirus positive cases were CV-A6 infections, highlighting the predominant role of CV-A6 in this outbreak. Interestingly, CV-A6 appeared sporadically since 2008 until it became the major genotype in 2012. It is noteworthy that CV-A6 was associated with HFMD and HA outbreaks in Japan [64,65], Finland [66,67], Singapore [53], Taiwan [68], China [69,70], Spain [71,72], and France [73] between 2005 and 2012.

We investigated the seroprevalence of EV71 in the Thai individuals using retrospective stored serum samples and the results showed high seropositive rates against EV71-B5 in individuals aged > 6 years (82.3% (71/86)). This suggests the presence of EV71 in Thailand during or before the time of sample collection. Results from epidemiological studies also revealed that EV71 infection was largely found in young children < 6 years of age, along with relatively low seropositive rates [74-76]. We found that the overall seropositive rate against EV71-B5 decreased by approximately 10% in 2011 and by 23% in 2012 compared to the rate in 2009 and 2010 (80%-84% seropositive rate). Therefore, the wide dissemination of viruses could have resulted in herd immunity. Our observation was also consistent with those of serosurveillance studies showing a similar seropositive pattern prior to the large outbreaks in China and Taiwan [77,78]. With respect to age, we further observed that infants had a high seropositive rate early in life (≤ 6 months; 66.7%), while more than 50% of children aged > 6 months to 2 years had low NAb against EV71-B5, indicating that NAb prevalence decreased with increasing age and thereafter remained constant in the older age groups. This rate was higher than the 50% observed in children > 6 years in Taiwan prior to the large outbreak in 2008 [78]. Furthermore, the age-specific GMT of EV71-B5 in childhood appeared to increase with age and was highest among primary school children aged > 6 years to 12 years, reflecting community-acquired virus transmission in those age groups. Evidence from serosurveillance studies also showed that immunity to one genotype is not cross-protective against another genotype [79]. Studies using series serum samples of parturient mothers and their healthy newborn infants indicated that the high proportion of herd immunity among susceptible children could have resulted from maternally transferred-NAb [74,80]. However, the passive maternal immunity to EV71 seems to be short-lived with approximately 42 days half-life, gradually decreases around the age of 4 months to 6 months and is undetectable at 12 months of age [74,81]. This could lead to the accumulation of individuals susceptible to EV71 infection, especially among older children, which may be responsible for the nationwide large outbreaks in many countries including Thailand. Based on the results from the cohort studies together with our results from Thai subjects, we recommend vaccination during the first year of life, particularly when infants are 6 months to 12 months of age.

Several studies have investigated the relationship between enterovirus types and their pathogenicity. In a study from the Netherlands, patients with EV1 genotype B infection were more likely to have neurologic complications than those infected with other genotypes [1]. However, studies from Taiwan and Malaysia demonstrated that there were no statistically significant differences in type variability between the different levels of clinical complication [11,82]. In this study, we found no significant differences in clinical complications caused by CV-A6 or other subgenotypes of EV71. We are aware that the prevalence of enteroviruses identified in Thailand may be underestimated, possibly due to the high proportion of underreported cases in children since infections often produce mild symptoms, self-limiting, and thus may not have received medical care. To enhance the sensitivity of the current system in Thailand, surveillance by both physicians and confirmed laboratory diagnosis is crucial to predict EV71 and other enterovirus outbreaks in the future. Hospital and healthcare staff in both the public and private sectors should either report unusual cases to the provincial health bureau or ask for assistance from the health ministry.

## Conclusions

Large epidemics of HFMD have never been documented in Thailand prior to 2012, yet the high seropositive rate of this study population suggests that the causative pathogen EV71 was already widely circulated in Thailand. Decreased EV71 circulation in early 2012 and in previous years might have increased the number of susceptible young children, which eventually resulted in the large outbreak in 2012. This is the first study to describe the seroepidemiology of EV71 in Thailand. Further work to support the hypothesis that natural EV71 transmission occurs largely among populations especially in preschool settings will require additional comprehensive serosurveillance studies.

## Competing interests

The authors have declared that no competing interests exist.

## Authors’ contributions

YP and JW conceptualized the project. PL designed the experiments and drafted the manuscript. PL, JP, SH, YW, and JM carried out the molecular studies. All authors read and approved the final manuscript.
